# 9G4 Autoreactivity Is Increased in HIV-Infected Patients and Correlates with HIV Broadly Neutralizing Serum Activity

**DOI:** 10.1371/journal.pone.0035356

**Published:** 2012-04-18

**Authors:** James J. Kobie, Danielle C. Alcena, Bo Zheng, Peter Bryk, Jonelle L. Mattiacio, Matthew Brewer, Celia LaBranche, Faith M. Young, Stephen Dewhurst, David C. Montefiori, Alexander F. Rosenberg, Changyong Feng, Xia Jin, Michael C. Keefer, Ignacio Sanz

**Affiliations:** 1 Division of Allergy, Immunology and Rheumatology, University of Rochester Medical Center, Rochester, New York, United States of America; 2 Department of Microbiology and Immunology, University of Rochester Medical Center, Rochester, New York, United States of America; 3 Department of Surgery, Laboratory for AIDS Vaccine Research and Development, Duke University Medical Center, Durham, North Carolina, United States of America; 4 J.P. Wilmot Cancer Center, University of Rochester Medical Center, Rochester, New York, United States of America; 5 Department of Biostatistics and Computational Biology, University of Rochester Medical Center, Rochester, New York, United States of America; 6 Division of Infectious Disease, University of Rochester Medical Center, Rochester, New York, United States of America; Emory University School of Medicine, United States of America

## Abstract

The induction of a broadly neutralizing antibody (BNAb) response against HIV-1 would be a desirable feature of a protective vaccine. Vaccine strategies thus far have failed to elicit broadly neutralizing antibody responses; however a minority of HIV-infected patients do develop circulating BNAbs, from which several potent broadly neutralizing monoclonal antibodies (mAbs) have been isolated. The findings that several BNmAbs exhibit autoreactivity and that autoreactive serum antibodies are observed in some HIV patients have advanced the possibility that enforcement of self-tolerance may contribute to the rarity of BNAbs. To examine the possible breakdown of tolerance in HIV patients, we utilized the 9G4 anti-idiotype antibody system, enabling resolution of both autoreactive VH4-34 gene-expressing B cells and serum antibodies. Compared with healthy controls, HIV patients had significantly elevated 9G4+ serum IgG antibody concentrations and frequencies of 9G4+ B cells, a finding characteristic of systemic lupus erythematosus (SLE) patients, both of which positively correlated with HIV viral load. Compared to the global 9G4−IgD− memory B cell population, the 9G4+IgD− memory fraction in HIV patients was dominated by isotype switched IgG+ B cells, but had a more prominent bias toward “IgM only" memory. HIV envelope reactivity was observed both in the 9G4+ serum antibody and 9G4+ B cell population. 9G4+ IgG serum antibody levels positively correlated (r = 0.403, p = 0.0019) with the serum HIV BNAbs. Interestingly, other serum autoantibodies commonly found in SLE (anti-dsDNA, ANA, anti-CL) did not correlate with serum HIV BNAbs. 9G4-associated autoreactivity is preferentially expanded in chronic HIV infection as compared to other SLE autoreactivities. Therefore, the 9G4 system provides an effective tool to examine autoreactivity in HIV patients. Our results suggest that the development of HIV BNAbs is not merely a consequence of a general breakdown in tolerance, but rather a more intricate expansion of selective autoreactive B cells and antibodies.

## Introduction

HIV infection is a major global health issue, and there is a critical need for a protective vaccine. The primary focus for humoral-mediated protection is the induction of neutralizing antibodies that recognize the HIV Envelope glycoprotein (Env). Although antibodies that recognize Env readily develop in HIV-1 -infected patients and can be induced by vaccination, these antibodies primarily recognize immunodominant, highly variable domains [1], consequently conferring little to no protection from the rapidly evolving virus. A minority of HIV patients develop serum antibodies that can neutralize a broad range of HIV isolates [2], [3], [4]. These broadly neutralizing antibodies (BNAbs) typically do not arise before three years post-infection [5], [6], and their occurrence correlates with viral load (VL) [2], [5], [7], suggesting that long-term antigen-driven evolution of the humoral response may be required for their development.

The limited incidence of persons producing HIV-reactive BNAbs in response to infection may in part result from proper enforcement of immunological tolerance for cross-reactive self-antigens. A relationship between autoreactive antibody and HIV BNAb development has been highlighted by several observations. In HIV patients, anti-CL serum antibodies correlate with increased HIV neutralization breadth [8], and several HIV broadly neutralizing monoclonal antibodies, including 2F5, 4E10, and 12A21 have been reported to have reactivity to self-antigens including dsDNA, insulin, Ro, histones, centromere B, and CL [9], [10], [11], although this still remains contentious [12], [13]. Additionally, many patients with connective tissue autoimmune disorders, including SLE and anti-phospholipid syndrome (APS), exhibit limited HIV neutralizing activity [14], [15]. Thus, during normal B cell development, a proportion of B cells with the potential to give rise to HIV BNAbs may be deleted or rendered anergic by engagement of corresponding self-antigen, and thus their development into mature B cells and antibody-secreting cells may require self-tolerance to be subverted. However, during HIV infection, substantial B cell hyperactivation manifested by polyclonal B cell activation and hypergammaglobulinemia [16], may contribute to disruption of tolerance, leading to the development of autoreactive antibodies in HIV patients, including those HIV BNAbs with autoreactivity. In addition to increases in autoantibodies including anti-CL, anti-dsDNA, anti-nuclear antibodies (ANA) and others in HIV patients, dramatic alterations in B cell homeostasis are reflected by the expansion of immature/transitional B cells, exhausted tissue like-memory B cells [17] and plasmablasts [18], and decreased resting memory and IgM memory [16], [19]. Many of these serological and cellular alterations are reversed with anti-retroviral therapy [16], suggesting they result from ongoing HIV viral replication.

Our group and others previously described an approach to monitor the development of autoreactive B cells and antibodies in SLE using the 9G4 anti-idiotype antibody [20], [21], [22], [23]. The rat anti-human monoclonal antibody 9G4 recognizes VH4-34 (previously designated VH4-21) -encoded antibodies and the B cells expressing these antibodies as surface receptor (heretofore referred to as 9G4+ antibodies and 9G4+ B cells respectively) [24]. Our laboratory has extensively studied the 9G4 system to understand human B cell tolerance and its breakdown in SLE [20], [25]. The advantage of the 9G4 system is predicated on a number of unique and helpful features: 1) 9G4+ antibodies are intrinsically autoreactive against N-acetylactosamine moietes expressed by blood group antigens as well as by multiple tissue antigens and glycoproteins [26], [27], [28], [29], including B220/CD45R which is expressed by many naïve B cells [26]. This reactivity is dependent on the heavy chain framework 1-encoded idiotope recognized by the 9G4 monoclonal antibody [24], [30]. Many 9G4+ antibodies also cross-react with glycolipids including LPS and gangliosides and in association with favorable HCDR3s (heavy chain complementary determining region 3), they may also recognize DNA [24], [31], [32]; 2) 9G4+ B cells, despite their autoreactivity, represent a sizable fraction (5–10%) of the normal repertoire in all healthy subjects; yet, they are strictly censored from the germinal centers (GC) and from expanding into the long-lived IgG memory and plasma cell compartments [33]; 3) such censoring accounts for the almost complete absence of serum 9G4+ antibodies in healthy subjects [20], [34]; 4) by contrast, GC censoring of 9G4+ B cells is faulty in SLE and results in large expansions of 9G4+ IgG memory and plasma cells in these patients [20], [34].

In this study, we utilized the 9G4 system to examine both autoreactive 9G4+ serum antibodies and B cells in HIV patients, and their relationship with clinical characteristics, HIV reactivity, and the occurrence of HIV BNAbs.

## Materials and Methods

### Clinical Samples

Peripheral blood samples were obtained from HIV-1 -infected patients at the University of Rochester Medical Center and the University of Washington HIV clinics between 2004 and 2010. Samples from healthy control (HC) subjects were obtained at the University of Rochester. All subjects provided signed written informed consent. Isolated peripheral blood mononuclear cells and serum were cryopreserved before subsequent analysis. All procedures and methods were approved by the University of Rochester Research Subjects Review Board and the University of Washington Institutional Review Board.

### Flow Cytometry

For global B cell phenotypic analysis peripheral blood mononuclear cells (PBMCs) were stained with anti-CD19-APC-Cy7 (SJ25C1, BD), anti-CD20-AlexaFluor 700 (2H7, Biolegend, San Diego, CA), anti-CD3-PerCP-Cy5.5 (SP34-2, BD), anti-IgD-FITC (IA6-2, BD), anti-IgG-PE (G18-145, BD), anti-IgM-PE-Cy5 (G20-127, BD), anti-CD27-Qdot655 (CLB-27/1, Invitrogen), 9G4-Pacific Blue, and Live/Dead fixable aqua dead cell stain (Invitrogen). The 9G4 mAb was kindly provided by F.K. Stevenson (University of Southhampton, Southhampton, United Kingdom), and recognizes a framework 1 region–encoded idiotype that is expressed by all unmutated, and close to 90% of mutated VH4-34 B cells present in the normal repertoire [35]–[36]. 9G4 painting of B cells was assessed using methods similar to those previously described [21], [26]. Briefly, cells were incubated at either 37**°**C or 4**°**C for 30 minutes in complete media (RPMI 1640+10% fetal bovine serum), and washed once with PBS, prior to staining with fluorochrome-conjugated antibodies at 4**°**C for 60 minutes. One-to-two million total events per sample were collected on an LSRII instrument (BD Biosciences) and analysis performed in a blinded manner using FlowJo software (Treestar, Inc, Ashland, OR). Total PBMC were gated on lymphocytes using FSC and SSC. Live/Dead stain and anti-CD3 were used to exclude dead cells and T cells, respectively.

To measure gp140-specific B cells, PBMC were first incubated for 30 minutes at 37**°**C to reduce 9G4 painting, then stained with purified oligomeric HIV-1 SF162 (clade B) and KNH1144 (clade A) gp140 directly conjugated to AlexaFluor660 and AlexaFluor 647, respectively and HIV-1 p24 (NIH AIDS Research and Reference Reagent Program) directly conjugated to AlexaFluor488, in addition to anti-CD19-PE-Cy7, anti-CD20-APC-Cy7, anti-IgD-PE, anti-IgM-PerCP-Cy5.5, anti-CD3-PE-Cy5, anti-CD14-PE-Cy5, 7AAD for dead cell exclusion, and biotinylated 9G4 mAb/streptavidin Qdot800 (Invitrogen) at 4**°**C for 60 minutes. At least one million total CD19+ B cells (≈10 million total PBMC) were analyzed per sample. p24+ gp140+ double-positive cells, a very rare population, were excluded from the analysis because they represent B cells that non-specifically react with gp140.

### ELISA

The detection of IgG 9G4+ Abs was performed as previously described [26]. Briefly, ELISA plates were coated with anti-human IgG (Jackson ImmunoResearch, West Grove, PA), blocked with 2% nonfat dry milk/2% BSA for 1 h at 37**°**C, then washed with 0.1% Tween 20 in PBS. Samples were serially diluted in triplicate in PBS containing 0.01% Tween 20 and 0.5% BSA, and incubated for 90 minutes at 37**°**C. Plates were washed, and binding was detected using biotinylated 9G4 mAb and streptavidin alkaline phosphatase. A human VH4-34 9G4-reactive mAb, 75D9, was used as a standard. For detection of gp140-reactive IgG and 9G4+ Abs, ELISA plates were coated with purified oligomeric HIV-1 YU2 gp140 [37] at 2 µg/ml and detected with either horseradish peroxidase conjugated anti-human IgG (Jackson ImmunoResearch) or biotinylated 9G4 Ab and streptavidin-HRP. To detect anti-CL IgG Abs, plates were coated with 50 µg/ml bovine cardiolipin (Sigma) in hexanes, and detected with anti-IgG HRP. For quantitative purposes the polyclonal human anti-human CL Ab (Lifespan Biosciences, Seattle, WA) was used at 1∶100 dilution, and assigned a relative units value of 1000, for which all serum samples were normalized to; positive/negative cutoff was determined as the upper 95% CI for serum from healthy control subjects. Anti-dsDNA IgG ELISA and Anti-Nuclear Antigen (ANA) IgG ELISA were performed, testing samples in duplicate using kits according to manufacturer's (Inova Diagnostics, Sand Diego, CA) recommendations.

### HIV-1 Neutralizing Activity

The neutralizing activity of sera was determined as previously described [38]. Briefly, a panel of clade B Tier 1 (SF162) and Tier 2 (6353.3, QH0692.42, SC422661.8, PVO.4, and AC10.0.29) pseudotyped viruses were incubated with heat-inactivated sera and then added to TZM-bl indicator cells. SVA-MLV (murine retrovirus Env-pseudovirus) was included as a negative control to confirm the absence of anti-retroviral therapy (ART). Neutralizing antibody titers were determined as the reciprocal serum dilution at which luciferase expression was reduced by 50% (ID50). To enable correlative analysis a single, composite BNAb score was determined using the geometric mean ID50 of the serum Tier 2 neutralizing activity, similar to that previously described [2], and if the ID50 was <20 for an individual virus, the value for that virus was set to 1 to enable calculation.

### Statistical Analysis

Two-tailed t test or Mann-Whitney test were used as appropriate to compare HC and HIV groups. Spearman two-tailed correlation co-efficient was used to measure the correlation of two variables. Statistical analyses were performed using Prism 5.0 software (GraphPad Software, La Jolla, CA) and significance was taken as p<0.05.

## Results

### HIV-infected patient population

Peripheral blood lymphocytes and serum samples were obtained from a diverse group of 90 HIV-1 seropositive patients from the University of Rochester and the University of Washington Center for AIDS Research (CFAR) cohorts. Most HIV patients (84%) had no history of anti-retroviral treatment (ART naïve) and the remainder were not receiving ART at time of sampling as per standard of care (**Table 1**). Samples from HIV-1 -negative healthy control (HC) subjects were obtained at the University of Rochester.

**Table 1:** HIV-1 patient demographics.

HIV patients (n)	90	range
**Age (YR)**	41.9+/−9.9	22–70
**Female (%)**	38.6	
**Time since HIV Dx (YR)**	6.3+/−5.6	0.1–23.3
**ART naïve (%)**	84.1	
**Non-Caucasian (%)**	51.2	
**Black (%)**	48.2	
**Viral load (copies/ml)**	35,500+/−111,629	45–942,000
**CD4 (cells/ml)**	549+/−237	7–1,080
	mean +/− SD indicated	

### HIV patients have elevated 9G4+ serum antibodies and 9G4+ B cells

**Figure 1:**
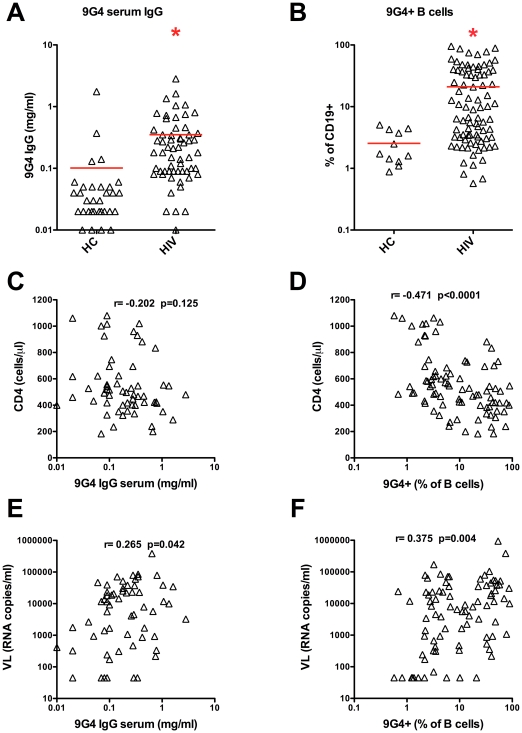
9G4+ serum antibodies and 9G4+ B cells are increased in HIV patients, correlating with CD4 and viral load. Peripheral blood was collected from ART-negative HIV patients and healthy control (HC) subjects, and serum and PBMC were isolated. **A.** 9G4+ serum IgG was determined by ELISA. **B.** The frequency of 9G4+ B cells was determined by flow cytometry. **C–F.** Spearmann correlation of 9G4+ serum IgG and 9G4+ B cells with CD4 and VL was determined for HIV patients. Each symbol represents a unique patient. * p<0.05 (Mann Whitney test).

Increased autoreactive antibodies are frequently observed in HIV patients, and therefore we asked if this breakdown in tolerance could be monitored using the 9G4 system as we and others have previously used for SLE [21], [26], [33], [39]. Indeed, HIV patients exhibited significant (p<0.01) and approximately 5-fold greater 9G4+ IgG serum antibody compared with HC subjects (**Fig. 1A**), with approximately 40% of HIV patients having 9G4+ IgG serum antibody levels exceeding the 95% CI of HC (not shown). HIV patients also exhibited a significant (p<0.05) 4-fold increase in the frequency of 9G4+ total peripheral blood B cells (**Fig. 1B**), with approximately 65% of HIV patients exceeding the 95% CI of HC (not shown). These results indicate that increased 9G4+ serum IgG and 9G4+ B cells are commonly found in many, but not all HIV patients, and are consistent with elevated autoreactive antibodies previously observed in HIV patients [8].

### 9G4+ antibody titers and 9G4+ B cell frequencies correlate with CD4 and viral load in HIV patients

On average, the HIV patients in our study had elevated 9G4+ IgG serum and 9G4+ B cells; however, a substantial continuum was observed (**Fig. 1**). To determine what factors might contribute to the development of 9G4+ IgG serum antibody and 9G4+ B cells in these patients, regression analysis was performed. Neither age nor time since HIV diagnosis correlated with 9G4+ serum IgG antibody or 9G4+ B cell frequency (data not shown). CD4+ T cell count negatively correlated with 9G4+ IgG serum (r = −0.202, p = 0.125) and 9G4+ B cells (r = −0.473, p<0.0001) (**Fig. 1C** **+D**), although only reached significance for 9G4+ B cells. HIV VL was significantly positively correlated with both 9G4+ IgG serum antibody (r = 0.265, p = 0.042) and 9G4+ B cells (r = 0.374, p = 0.004) (**Fig. 1E** **+F**). Interestingly, HIV VL did not correlate with the abundance of other autoreactive serum antibodies including anti-dsDNA, ANA, or anti-CL (**Fig S1**). These results suggest that HIV viral replication may selectively promote the development of autoreactive 9G4+ B cells and 9G4+ serum antibody.

### Phenotype of 9G4+ B cells in HIV patients

**Figure 2:**
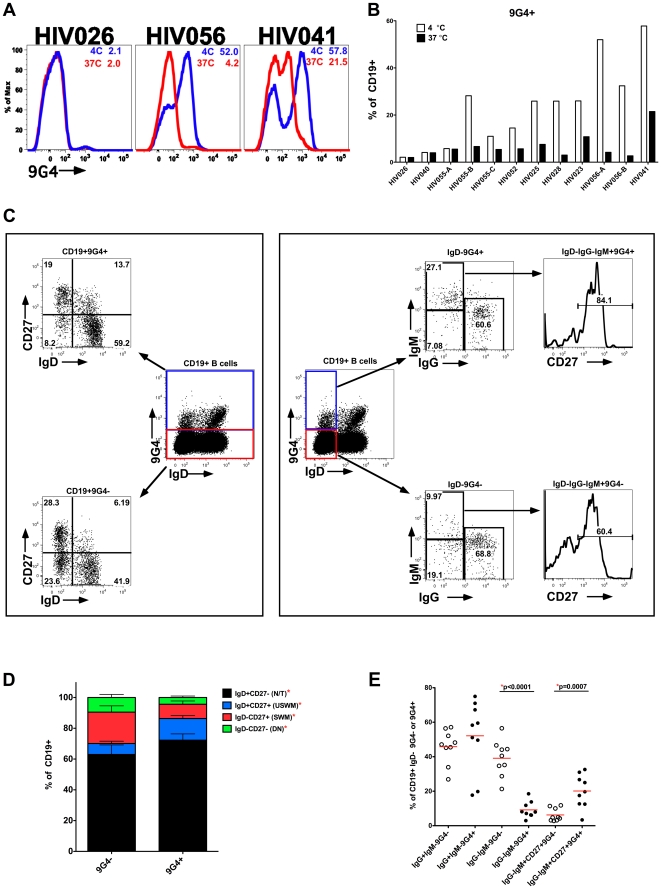
Phenotype of 9G4+ B cells in HIV patients. PBMC were incubated at 4**°**C or 37**°**C for 30 minutes prior to incubation at 4**°**C with antibodies for flow cytometric analysis. **A.** Representative B cell 9G4 expression profiles. Inset numbers represent frequency of 9G4+ B cells among total CD19+ B cells. **B.** 9G4+ B cell frequency for multiple samples; -A, -B, -C, indicate consecutive longitudinal samples from the same HIV patient. **C–E.** 9G4+ and 9G4− B cells incubated at 37°C for 30 minutes and then stained for surface markers and phenotypic analysis peformed using indicated gating strategy (**C**) to determine composition of total CD19+ total B cell population (**D**) and IgD−CD19+ memory B cell population (**E**) determined for 9 samples from unique HIV patients. * p<0.05 (two-tailed paired t-test).

It is striking that for many HIV patients, greater than 50% of their total B cell compartment is 9G4+. We previously reported that in SLE, 9G4+ serum antibodies can bind to B220, which is expressed on the surface of naïve B cells and some memory B cells [26]. This high frequency of 9G4+ B cells in some of the HIV patients may result from measuring both the B cells actually expressing VH4-34-encoded BCR, and also the indirect binding of the 9G4 reagent to serum VH4-34-encoded autoreactive antibody bound to B220 on the B cell, as previously described in SLE patients and not HC subjects [21], [26] and heretofore referred to as painting. To determine if 9G4 painting of B cells may be occurring in HIV patients, samples were incubated at 37**°**C to dissociate bound 9G4+ serum antibody prior to 9G4 reagent staining at 4**°**C. This approach revealed that similar to SLE [21], [26], 9G4 painting does occur in some HIV patients (**Fig. 2A** **+B**), and that soluble autoreactive 9G4+ serum antibody is bound to a substantial proportion of the total B cells. Interestingly, limited longitudinal sampling (HIV055 and HIV056) indicated that 9G4 painting may substantially fluctuate over time thereby suggesting episodic stimulation of 9G4 antibodies rather than steady production through long-lived plasma cells or continuous B cell stimulation (**Fig. 2B**).

By eliminating 9G4 painting, the composition of the actual 9G4+ B cell population in HIV patients was determined (**Fig. 2C**). The 9G4+ B cell population in HIV patients was comprised of significantly greater frequencies of IgD+CD27− naïve/transitional (N/T) and IgD+CD27+ unswitched memory B cells (USWM), and significantly lower frequencies of IgD−CD27+ switched memory (SWM) B cells and IgD−CD27− double negative (DN) B cells as compared with the 9G4− B cell population (**Fig. 2D**). This observation suggests that 9G4+ B cells may continue to be partially censored from IgD-memory compartments in HIV patients, or may reflect the remaining influence of pre-HIV infection 9G4+ censorship on memory compartment composition.

Because 9G4+ B cells are rare in the IgD− memory compartment in HC (**Fig S2**) [26], [33], we infer that IgD−9G4+ memory B cells in HIV patients developed in response to HIV infection and therefore their composition was further assessed. Within both the 9G4+ and 9G4− fractions of the IgD− memory B cell compartment, IgG+ memory cells predominated (**Fig. 2E**), at 52% and 46% respectively. Interestingly, within the IgD−9G4+ memory compartment, “IgM only" memory (IgG−IgM+CD27+) was a substantial component compared with the composition of the IgD−9G4− memory compartment, 20% and 6% respectively, and this difference was significant (p = 0.0007). Conversely, a more prominent IgM−IgG− population, likely the IgA memory population, was evident in the IgD−9G4− compartment, compared to the IgD−9G4+ compartment, 39% and 9% respectively, and this difference was significant (p<0.0001). These differences in the composition of 9G4+ and 9G4− IgD− memory compartment may indicate distinct developmental pathways by which 9G4+ memory B cells cell arise.

### HIV Env reactivity of 9G4+ serum antibody and 9G4+ B cells from HIV patients

**Figure 3:**
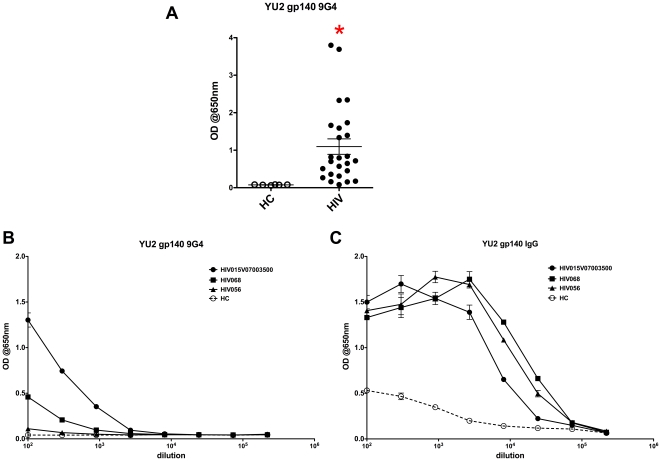
Detection of 9G4+ gp140-reactive serum antibodies. **A.** Serum was diluted 1∶100 and 9G4+ YU2 gp140-reactive antibody detected by ELISA. Each symbol represents a unique patient. *p<0.05. **B–C.** Serum from 3 HIV patients and 1 HC subject was serially diluted and 9G4+ gp140-reactive (**B**) and total IgG gp140-reactive (**C**) antibodies determined, and mean +/− SEM for assay triplicates presented.

Observing the positive correlation of HIV VL and 9G4+ serum IgG, and considering the autoreactivity of several well-characterized HIV Env-specific monoclonal antibodies, we next sought to determine if 9G4+ serum antibodies are reactive to HIV Env. YU2gp140-reactive 9G4+ serum antibodies were detected in many HIV patients (**Fig. 3A**), although not surprisingly, these antibodies likely represent only a minority of total YU2gp140-reactive serum antibodies in HIV patients (**Fig. 3B** **+3C**). However, the amount of Env-reactive 9G4+ serum antibody may be underestimated by this approach, because binding to Env may be mediated in part through the specific VH4-34 FR1 idiotope recognized by the 9G4 monoclonal antibody, thus blocking its detection.

**Figure 4:**
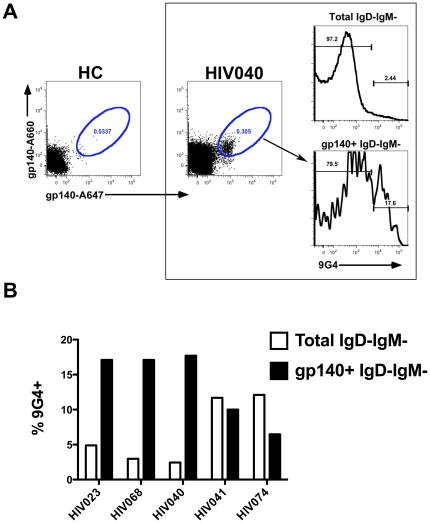
Identification of 9G4+ gp140-reactive B cells. PBMC were incubated at 37**°**C for 30 minutes, then stained at 4**°**C with fluorescently conjugated gp140 and antibodies. **A.** Representative flow profile of a HC and HIV sample (HIV040). Plots are gated on live, CD14−CD3−CD19+IgD−IgM−p24− B cells. **B.** The frequency of 9G4+ B cells within the total IgD− IgM− B cell subset or gp140+ IgD−IgM− B cell subset is indicated.

We also conducted Env-specific flow cytometry to identify Env-reactive 9G4+ memory B cells in HIV patients. 9G4+ Env-reactive IgD−IgM− memory B cells were present in HIV patients (**Fig. 4**). A mean of ≈14% of the Env-reactive IgD−IgM− memory B cells were 9G4+, whereas 9G4+ cells comprised a mean of only ≈7% of the total IgD−IgM− memory B cells (**Fig. 4B**). Although this difference was not statistically significant, it does suggest a selective expansion of 9G4+ B cells within the Env-reactive B cell repertoire in some patients. The presence of Env-reactive 9G4+ B cells was also confirmed by memory B cell EliSpot (**Fig S3**). These results suggest that HIV infection promotes the expansion of 9G4+ B cells and antibodies, a proportion of which are Env-reactive.

### 9G4+ serum antibody and 9G4+ B cells correlate with serum HIV broadly neutralizing activity

Given the auto- and Env-reactive properties of 9G4+ antibodies and B cells, and the rationale that impaired tolerance and autoreactivity may be advantageous for developing a broadly neutralizing response against HIV, we assessed the magnitude and breadth of serum neutralizing activity against a panel of Tier 1 and Tier 2 clade B isolates (**Table S1A**). Multi-clade neutralization was assessed on select samples (**Table S1B**). To facilitate regression analysis, we used a composite broadly neutralizing activity measure, the geometric mean ID50 of the serum Tier 2 clade B neutralizing activity, similar to that previously described [2]. The samples showed a diverse range of neutralizing activity, including patients HIV073 and HIV026 (geomean ID50 = 1555 and 615, respectively), which represent, approximately the top 2% of all HIV samples tested by the Montefiori laboratory.

**Figure 5:**
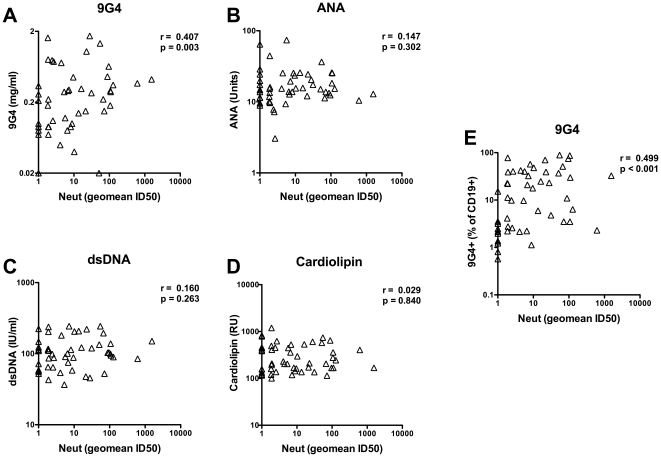
9G4+ serum antibodies and 9G4+ B cells correlate with HIV serum broadly neutralizing activity. HIV neutralizing activity of serum against a panel of five Tier II isolates was determined by TZMbl assay and geometric mean ID50 presented. Serum IgG reactive to 9G4 (**A**), ANA (**B**), dsDNA (**C**) and cardiolipin (**D**) was determined by ELISA. **E.** The frequency of 9G4+ B cells was determined by flow cytometry. Spearmann correlation indicated. Each symbol represents a unique patient.

A significant positive correlation (r = 0.407, p = 0.003) was observed between 9G4+ IgG serum antibody and HIV neutralizing activity (**Fig. 5A**). Interestingly, anti-dsDNA, ANA, and anti-CL serum antibodies did not correlate with HIV neutralizing activity (**Fig. 5B–D**). The frequency of 9G4+ B cells also positively correlated (r = 0.499, p<0.001) with HIV neutralizing activity (**Fig. 5E**). Notably, HIV neutralizing activity also significantly correlated with HIV VL (r = 0.411, p = 0.003) (**Fig S4**) consistent with previous findings [2], [5], [7]. These results suggest that BNAb development may not be caused by an overall breakdown in tolerance, but rather a more specific subversion of tolerance mechanisms, evident by the expansion of 9G4+ B cells and antibodies.

## Discussion

This study of ART-negative HIV patients demonstrated dramatically increased production of 9G4+ IgG serum antibodies (**Fig. 1A**) and the *in vivo* autoreactivity of these 9G4 antibodies as indicated by their binding to B cells (**Fig. 2A** **+B**). This increase was positively correlated with HIV VL (**Fig. 1E**). There was also a significant expansion of 9G4+ memory B cells that had distinct phenotypes compared with 9G4− memory B cells as indicated by IgG+ and IgM+ memory B cell populations being over-represented in the 9G4+ memory compartments (**Fig. 2C** **+D**). A fraction of the 9G4+ serum antibodies and B cells were reactive to HIV Env (**Fig. 3** **+4**), and the abundance of 9G4+ serum antibodies and B cells positively correlated with serum HIV neutralizing activity (**Fig. 5**). These findings demonstrate the association of autoreactivity with the development of HIV BNAb at both the cellular level and at the level of serum antibodies.

The presence of 9G4+ IgG serum antibody and 9G4+ memory B cells is consistent with the impaired B cell tolerance in HIV patients which has been previously observed, primarily as autoreactive serum antibodies [8], [40], [41]. Although the presence of elevated 9G4+ IgG serum appears to be slightly greater (40%) as compared to anti-dsDNA, ANA, and CL (15%, 24%, 31% respectively) in our cohort. Furthermore, we observed a significant positive correlation between HIV VL and 9G4+ IgG antibody and 9G4+ B cells, but no significant correlation between VL and dsDNA, ANA, or CL reactive IgG antibodies. This suggests that 9G4 induction may be a more selective process induced by the HIV virus, either directly through viral antigens or indirectly through the generation of autoantigens known to react with 9G4+ antibodies, such as apoptotic cells [42], [43], as opposed to simply generalized B cell activation which might, at least in part, lead to the development of other autoreactivities in HIV patients, such as anti-DNA and anti-CL antibodies. The former possibility is being currently addressed by the characterization of 9G4 monoclonal antibodies generated from HIV patients using single-cell methodologies, and will additionally enable the assessment of polyreactivity within the 9G4 compartment. The role of the virus in the induction of the 9G4 responses could also be clarified in part by ongoing longitudinal analysis of HIV patients, before and after the initiation of ART, or starting during acute infection to examine the initial emergence of the 9G4 response, however, this is beyond the scope of the current study.

Although 9G4+ B cells shared many phenotypic commonalities with 9G4− B cells in HIV patients, including their presence in both naïve and memory B cell compartments, they were over-represented in the “IgM only" memory (IgD−IgG−IgM+CD27+) compartment compared to 9G4− B cells (**Fig. 2D**). The rare IgM only memory population, is expanded in patients deficient in activation-induced cytidine deaminase (AID) [44] and has been suggested to result from cells exiting prematurely from the GC reaction before class switching occurs or may be due to the result of GC-independent B cell development [45], [46]. This would be consistent with the features of 9G4+ B cell regulation, where in healthy control subjects, they are fully excluded from the GC. However in HIV patients, some 9G4+ B cells are likely to have participated fully in GC reactions, as evidenced by IgD−IgG+ 9G4+ B cell populations. Other 9G4+ B cells in HIV patients may still be subject to intrinsic or extrinsic regulation that prevents full GC participation, as evidenced by the over-representation of the IgM only memory population within the 9G4+ B cell compartment. It is tantalizing to speculate on properties that may distinguish 9G4+ IgM only memory vs. 9G4+ IgD−IgM−IgG+ memory B cells, such as degree of autoreactivity, responsiveness to T cell help or suppression, or capacity to migrate toward CXCL13. These possibilities will be dissected in future studies. Additional phenotypic differences in the 9G4+ B cell population may exist and may be revealed by more comprehensive flow-cytometric based profiling.

The observation of a higher frequency of 9G4+ cells in the Env-specific memory B cell population compared with the total memory population suggests in some HIV patients antigen-specific 9G4+ expansion may be occurring. This observation warrants further investigation to determine if such patients have unique features, and to address the possible mechanism of 9G4+ Env-specific memory B cell expansion. The positive correlation observed between serum HIV neutralizing activity and 9G4+ IgG may reflect a direct contribution of 9G4+ IgG to HIV neutralizing activity, or it might reflect an immunological environment, in which HIV infection coincidently favors the expansion of both species. It is evident that 9G4+ cells and serum antibodies represent only a minority of the Env-specific repertoire, but it remains to be determined if 9G4+ Env-specific cells and antibodies have any distinct properties beyond inherent autoreactivity that may uniquely impact HIV. Although numerous HIV broadly neutralizing monoclonal antibodies have been described to date, none are VH4-34-encoded. This does not preclude their existence or the potential of 9G4+ B cells and antibodies to contribute substantially to a protective immune response to HIV. Indeed, given the reactivity 9G4+ antibodies exhibit for glycoproteins, including an N-linked N acetyllactosamine determinant on CD45R/B2220 [26] and the I/I blood group antigen [27], [28], [29], reactivity of 9G4+ antibodies to HIV Env may in part be mediated through interaction with glycosylated epitopes. Thus, detailed assessment of the 9G4+ serum antibodies for HIV neutralizing activity and specificity should be pursued in future studies. The expansion of 9G4 in HIV patients could also result from the polyclonal B cell activation that is readily observed in HIV patients, which may result from numerous factors including direct interaction of HIV and B cells through CD21, DC-SIGN, TLR7, and TLR9 and also indirectly as a consequence of cytokine upregulation (e.g. IL4, IL10, IL6, IFNα) and T cell help [47].

This work demonstrates the utility of the 9G4 system in interrogating autoreactivity at the cellular level in HIV patients, enabling insight into the development and expansion of autoreactive B cells and their relationship to the serum antibody repertoire. Important future questions include whether autoreactive antibodies are a prerequisite for the development of a protective HIV humoral response. There are clear examples of individual BNAbs against HIV that have minimal autoreactivity (e.g. VRC01) [48], but it is unclear if there is a role for relaxed tolerance in promoting their development. And although it is likely that autoreactivity in HIV patients may be in part a consequence of B cell polyclonal activation, polyclonal activation may also greatly expand the available repertoire of HIV-specific B cells. For example, a hyperactive state of global B cell dysregulation and functional threshold decreases may allow B cells that bind only weakly to HIV-1 Env to productively respond to this viral antigen, and give rise to antibody-secreting cells.

The association of autoreactivity and BNAbs also has important implications for HIV vaccine development – especially if autoreactivity is indeed a prerequisite for the development of a subset of immunoglobulin specificities that have the potential to give rise to BNAbs. Although it may not be feasible to completely uncouple autoreactivity from BNAb development in a vaccine setting, it is important to note that autoreactivity does not necessarily equate to manifestations of chronic autoimmune pathology, or a definitive pre-disposition to autoimmune disease development. Several observations support this, including the development of “non-pathological" autoreactive antibodies during various viral infections including CMV, HCV, and RSV [49], [50], [51]. Consistent with this, a number of investigators have described autoreactive antibodies in HIV-infected individuals as ‘non-pathogenic", based on distinct reactivity profiles [13] or lack of β2GP1 involvement [52] - as compared with HIV-negative patients with primary autoimmune diseases. Indeed, autoreactive antibodies are frequently observed in otherwise normal healthy individuals, absent of any clinical autoimmune disease manifestations. Minimally, the association of auto- and HIV- reactivity warrants detailed examination, which can be facilitated by the 9G4 system, as it may reveal critical cellular and molecular mechanisms by which the repertoire of potential BNAb specificities is effectively engaged and stimulated to develop into long-lived plasma cells conferring sustained protection from HIV infection.

## Supporting Information

Figure S1 **Common autoantibodies do not correlate with HIV viral load.** Serum samples from ART-negative HIV patients were assessed for IgG antibody reactive to ANA, dsDNA, and CL by ELISA. Plasma VL was determined by PCR at the same timepoint. Dotted line represents positive/negative cut-off value. Spearmann correlation indicated, each symbol represents a unique patient.
(TIFF)

Figure S2 **Rarity of 9G4+ B cells in the IgD− compartment of healthy control subjects.** PBMC were analyzed *ex vivo* by flow cytometry. **A.** Representative plots from a HC and HIV patient gated on total CD19+ B cells. **B.** The frequency of 9G4+ B cells within the IgD− compartment. Each symbol represents a unique patient. * p<0.05 (Mann Whitney test).
(TIF)

Figure S3 **Detection of 9G4+ IgG+ gp140 reactive memory B cells.** PBMC were obtained from three HIV patients and IgG+ B cells were isolated and cultured with CpG+IL-2 for 4 days for the generation of antibody-secreting cells. EliSpots were performed to identify total IgG, total 9G4, total IgG gp140, and 9G4+ gp140 specific antibody-secreting cells. EliSpot coating/detection antibody combinations indicated.
(TIF)

Figure S4 **Correlation of HIV serum broadly neutralizing activity and viral load.** HIV neutralizing activity of serum against a panel of five Tier II isolates was determined by TZMbl assay and geometric mean ID50 presented with HIV VL. Spearmann correlation indicated, each symbol represents a unique patient.
(TIFF)

Table S1 **HIV serum neutralizing activity.** **A.** Clade B serum neutralizing activity **B.** Multi-clade serum neutralizing activity.
(PDF)

## References

[1] Tomaras GD, Haynes BF (2009). HIV-1-specific antibody responses during acute and chronic HIV-1 infection.. Current opinion in HIV and AIDS.

[2] Doria-Rose NA, Klein RM, Daniels MG, O'Dell S, Nason M (2010). Breadth of human immunodeficiency virus-specific neutralizing activity in sera: clustering analysis and association with clinical variables.. J Virol.

[3] Sather DN, Armann J, Ching LK, Mavrantoni A, Sellhorn G (2009). Factors associated with the development of cross-reactive neutralizing antibodies during human immunodeficiency virus type 1 infection.. J Virol.

[4] Simek MD, Rida W, Priddy FH, Pung P, Carrow E (2009). Human immunodeficiency virus type 1 elite neutralizers: individuals with broad and potent neutralizing activity identified by using a high-throughput neutralization assay together with an analytical selection algorithm.. J Virol.

[5] Mikell I, Sather DN, Kalams SA, Altfeld M, Alter G (2011). Characteristics of the earliest cross-neutralizing antibody response to HIV-1.. PLoS Path.

[6] van Gils MJ, Euler Z, Schweighardt B, Wrin T, Schuitemaker H (2009). Prevalence of cross-reactive HIV-1-neutralizing activity in HIV-1-infected patients with rapid or slow disease progression.. AIDS.

[7] Piantadosi A, Panteleeff D, Blish CA, Baeten JM, Jaoko W (2009). Breadth of neutralizing antibody response to human immunodeficiency virus type 1 is affected by factors early in infection but does not influence disease progression.. J Virol.

[8] Gray ES, Taylor N, Wycuff D, Moore PL, Tomaras GD (2009). Antibody specificities associated with neutralization breadth in plasma from human immunodeficiency virus type 1 subtype C-infected blood donors.. J Virol.

[9] Haynes BF, Fleming J, St Clair EW, Katinger H, Stiegler G (2005). Cardiolipin polyspecific autoreactivity in two broadly neutralizing HIV-1 antibodies.. Science.

[10] Verkoczy L, Kelsoe G, Moody MA, Haynes BF (2011). Role of immune mechanisms in induction of HIV-1 broadly neutralizing antibodies.. Curr Opin Immunol.

[11] Scheid JF, Mouquet H, Ueberheide B, Diskin R, Klein F (2011). Sequence and Structural Convergence of Broad and Potent HIV Antibodies That Mimic CD4 Binding..

[12] Scherer EM, Zwick MB, Teyton L, Burton DR (2007). Difficulties in eliciting broadly neutralizing anti-HIV antibodies are not explained by cardiolipin autoreactivity.. AIDS.

[13] Singh H, Henry KA, Wu SS, Chruscinski A, Utz PJ (2011). Reactivity profiles of broadly neutralizing anti-HIV-1 antibodies are distinct from those of pathogenic autoantibodies.. AIDS.

[14] Scherl M, Posch U, Obermoser G, Ammann C, Sepp N (2006). Targeting human immunodeficiency virus type 1 with antibodies derived from patients with connective tissue disease.. Lupus.

[15] Douvas A, Takehana Y, Ehresmann G, Chernyovskiy T, Daar ES (1996). Neutralization of HIV type 1 infectivity by serum antibodies from a subset of autoimmune patients with mixed connective tissue disease.. AIDS Res Hum Retroviruses.

[16] Moir S, Fauci AS (2009). B cells in HIV infection and disease.. Nat Rev Immunol.

[17] Moir S, Ho J, Malaspina A, Wang W, DiPoto AC (2008). Evidence for HIV-associated B cell exhaustion in a dysfunctional memory B cell compartment in HIV-infected viremic individuals.. J Exp Med.

[18] Doria-Rose NA, Klein RM, Manion MM, O'Dell S, Phogat A (2009). Frequency and phenotype of human immunodeficiency virus envelope-specific B cells from patients with broadly cross-neutralizing antibodies.. J Virol.

[19] Morrow M, Valentin A, Little R, Yarchoan R, Pavlakis GN (2008). A Splenic Marginal Zone-Like Peripheral Blood CD27+B220 B Cell Population Is Preferentially Depleted in HIV Type 1-Infected Individuals.. AIDS Res Hum Retroviruses.

[20] Cappione A, III, Anolik JH, Pugh-Bernard A, Barnard J, Dutcher P (2005). Germinal center exclusion of autoreactive B cells is defective in human systemic lupus erythematosus.. J Clin Invest.

[21] Bhat NM, Lee LM, van Vollenhoven RF, Teng NN, Bieber MM (2002). VH4-34 encoded antibody in systemic lupus erythematosus: effect of isotype.. The Journal of rheumatology.

[22] Stevenson FK, Smith GJ, North J, Glennie MG, Hamblin TJ (1988). Use of a cross reacting anti-idiotype to identify normal counterparts of neoplastic cells.. Nouvelle revue francaise d'hematologie.

[23] Stevenson FK, Longhurst C, Chapman CJ, Ehrenstein M, Spellerberg MB (1993). Utilization of the VH4-21 gene segment by anti-DNA antibodies from patients with systemic lupus erythematosus.. J Autoimmun.

[24] Potter KN, Li Y, Pascual V, Williams RC, Byres LC (1993). Molecular characterization of a cross-reactive idiotope on human immunoglobulins utilizing the VH4-21 gene segment.. The Journal of experimental medicine.

[25] Milner EC, Anolik J, Cappione A, Sanz I (2005). Human innate B cells: a link between host defense and autoimmunity?. Springer Semin Immunopathol.

[26] Cappione AJ, Pugh-Bernard AE, Anolik JH, Sanz I (2004). Lupus IgG VH4.34 antibodies bind to a 220-kDa glycoform of CD45/B220 on the surface of human B lymphocytes.. J Immunol.

[27] Sanz I, Kelly P, Williams C, Scholl S, Tucker P (1989). The smaller human VH gene families display remarkably little polymorphism.. The EMBO journal.

[28] Borretzen M, Chapman C, Stevenson FK, Natvig JB, Thompson KM (1995). Structural analysis of VH4-21 encoded human IgM allo- and autoantibodies against red blood cells.. Scand J Immunol.

[29] Pascual V, Victor K, Lelsz D, Spellerberg MB, Hamblin TJ (1991). Nucleotide sequence analysis of the V regions of two IgM cold agglutinins. Evidence that the VH4-21 gene segment is responsible for the major cross-reactive idiotype.. J Immunol.

[30] Potter KN, Hobby P, Klijn S, Stevenson FK, Sutton BJ (2002). Evidence for involvement of a hydrophobic patch in framework region 1 of human V4-34-encoded Igs in recognition of the red blood cell I antigen.. J Immunol.

[31] Bhat NM, Bieber MM, Chapman CJ, Stevenson FK, Teng NN (1993). Human antilipid A monoclonal antibodies bind to human B cells and the i antigen on cord red blood cells.. J Immunol.

[32] van Es JH, Gmelig Meyling FH, van de Akker WR, Aanstoot H, Derksen RH (1991). Somatic mutations in the variable regions of a human IgG anti-double-stranded DNA autoantibody suggest a role for antigen in the induction of systemic lupus erythematosus.. The Journal of experimental medicine.

[33] Cappione A, 3rd, Anolik JH, Pugh-Bernard A, Barnard J, Dutcher P (2005). Germinal center exclusion of autoreactive B cells is defective in human systemic lupus erythematosus.. J Clin Invest.

[34] Pugh-Bernard AE, Silverman GJ, Cappione AJ, Villano ME, Ryan DH (2001). Regulation of inherently autoreactive VH4-34 B cells in the maintenance of human B cell tolerance.. J Clin Invest.

[35] Zheng NY, Wilson K, Wang X, Boston A, Kolar G (2004). Human immunoglobulin selection associated with class switch and possible tolerogenic origins for C delta class-switched B cells.. The Journal of clinical investigation.

[36] Mockridge CI, Rahman A, Buchan S, Hamblin T, Isenberg DA (2004). Common patterns of B cell perturbation and expanded V4-34 immunoglobulin gene usage in autoimmunity and infection.. Autoimmunity.

[37] Mattiacio J, Walter S, Brewer M, Domm W, Friedman AE (2011). Dense display of HIV-1 envelope spikes on the lambda phage scaffold does not result in the generation of improved antibody responses to HIV-1 Env.. Vaccine.

[38] Mascola JR, D'Souza P, Gilbert P, Hahn BH, Haigwood NL (2005). Recommendations for the design and use of standard virus panels to assess neutralizing antibody responses elicited by candidate human immunodeficiency virus type 1 vaccines.. J Virol.

[39] Bhat NM, Bieber MM, Yang YC, Leu YS, van Vollenhoven RF (2004). B cell lymphoproliferative disorders and VH4-34 gene encoded antibodies.. Hum Antibodies.

[40] Petrovas C, Vlachoyiannopoulos PG, Kordossis T, Moutsopoulos HM (1999). Anti-phospholipid antibodies in HIV infection and SLE with or without anti-phospholipid syndrome: comparisons of phospholipid specificity, avidity and reactivity with beta2-GPI.. J Autoimmun.

[41] Dennison SM, Anasti K, Scearce RM, Sutherland L, Parks R (2011). Nonneutralizing HIV-1 gp41 envelope cluster II human monoclonal antibodies show polyreactivity for binding to phospholipids and protein autoantigens.. J Virol.

[42] Catera R, Silverman GJ, Hatzi K, Seiler T, Didier S (2008). Chronic lymphocytic leukemia cells recognize conserved epitopes associated with apoptosis and oxidation.. Mol Med.

[43] Jenks SA, Palmer E, Marin E, Sanz I (2010). 9G4 Autoantibodies Dominate the Anti-Apoptotic Cell Autoimmune Response in SLE..

[44] Weller S, Braun MC, Tan BK, Rosenwald A, Cordier C (2004). Human blood IgM “memory" B cells are circulating splenic marginal zone B cells harboring a prediversified immunoglobulin repertoire.. Blood.

[45] Klein U, Kuppers R, Rajewsky K (1993). Human IgM+IgD+ B cells, the major B cell subset in the peripheral blood, express V kappa genes with no or little somatic mutation throughout life.. Eur J Immunol.

[46] Klein U, Kuppers R, Rajewsky K (1994). Variable region gene analysis of B cell subsets derived from a 4-year-old child: somatically mutated memory B cells accumulate in the peripheral blood already at young age.. The Journal of experimental medicine.

[47] Haas A, Zimmermann K, Oxenius A (2011). Antigen-Dependent and -Independent Mechanisms of T and B Cell Hyperactivation during Chronic Hiv-1 Infection..

[48] Zhou T, Georgiev I, Wu X, Yang ZY, Dai K (2010). Structural basis for broad and potent neutralization of HIV-1 by antibody VRC01.. Science.

[49] Zachou K, Liaskos C, Rigopoulou E, Gabeta S, Papamichalis P (2006). Presence of high avidity anticardiolipin antibodies in patients with autoimmune cholestatic liver diseases.. Clin Immunol.

[50] Forster J, Maier O, Lobbert J, Kaufmehl K, Streckert HJ (1996). Prevalence of antibodies against HEp-2 cell antigen in infants and children hospitalized with respiratory syncytial virus infection.. Infection.

[51] Uthman IW, Gharavi AE (2002). Viral infections and antiphospholipid antibodies.. Semin Arthritis Rheum.

[52] Martinez V, Diemert MC, Braibant M, Potard V, Charuel JL (2009). Anticardiolipin antibodies in HIV infection are independently associated with antibodies to the membrane proximal external region of gp41 and with cell-associated HIV DNA and immune activation.. Clinical infectious diseases : an official publication of the Infectious Diseases Society of America.

