# Supplementary figures and images for: 9G4 Autoreactivity Is Increased in HIV-Infected Patients and Correlates with HIV Broadly Neutralizing Serum Activity
Source: PLoS One. 2012 Apr 18;7(4):e35356. doi: 10.1371/journal.pone.0035356 (PMC3329433; doi:10.1371/journal.pone.0035356)

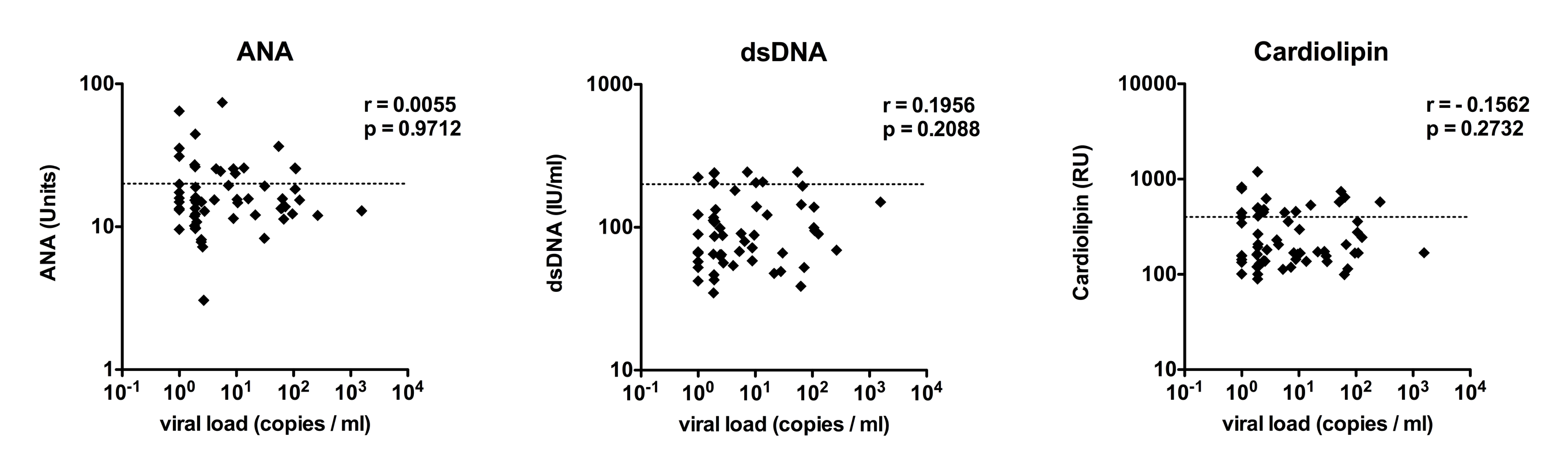

Supplement: Figure S1 — Common autoantibodies do not correlate with HIV viral load. Serum samples from ART-negative HIV patients were assessed for IgG antibody reactive to ANA, dsDNA, and CL by ELISA. Plasma VL was determined by PCR at the same timepoint. Dotted line represents positive/negative cut-off value. Spearmann correlation indicated, each symbol represents a unique patient. (TIFF) [file pone.0035356.s001.tiff]

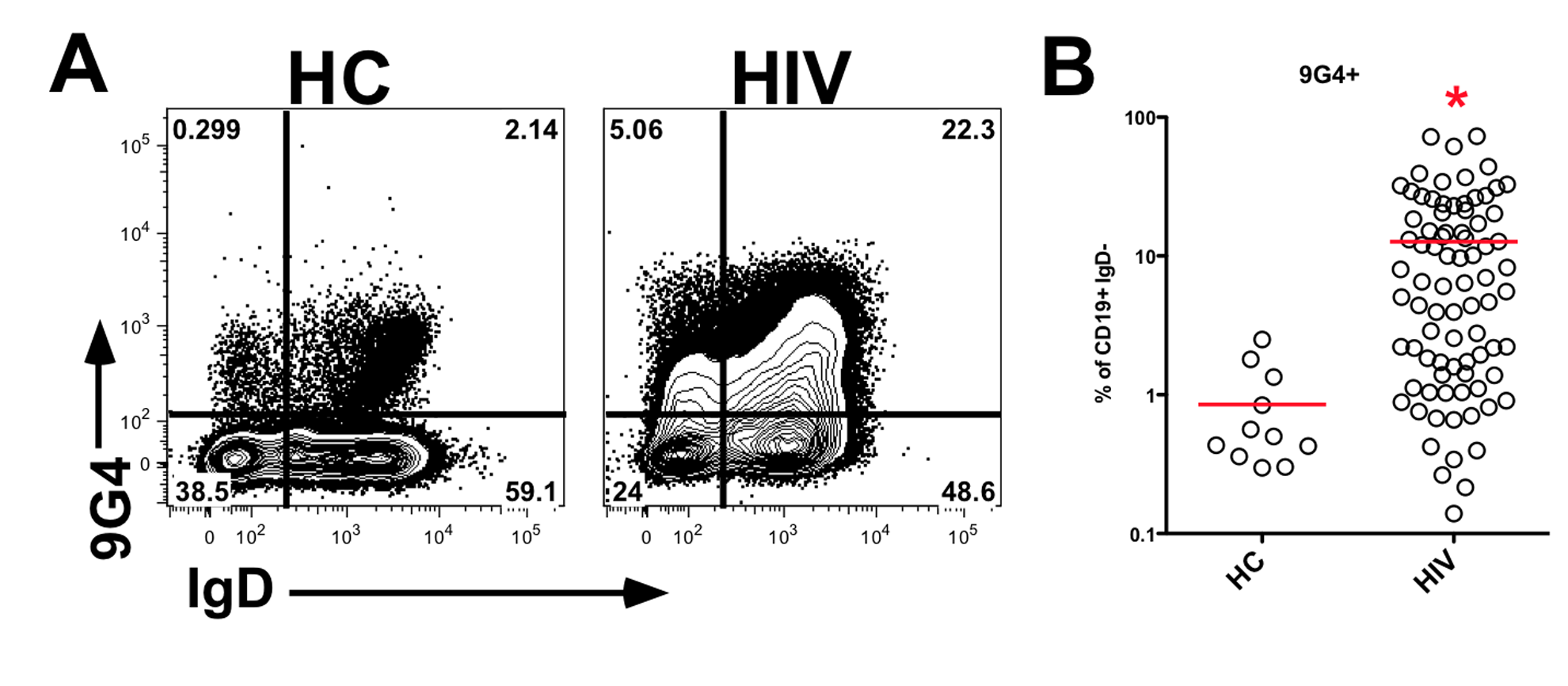

Supplement: Figure S2 — Rarity of 9G4+ B cells in the IgD− compartment of healthy control subjects. PBMC were analyzed ex vivo by flow cytometry. A. Representative plots from a HC and HIV patient gated on total CD19+ B cells. B. The frequency of 9G4+ B cells within the IgD− compartment. Each symbol represents a unique patient. * p<0.05 (Mann Whitney test). (TIF) [file pone.0035356.s002.tif]

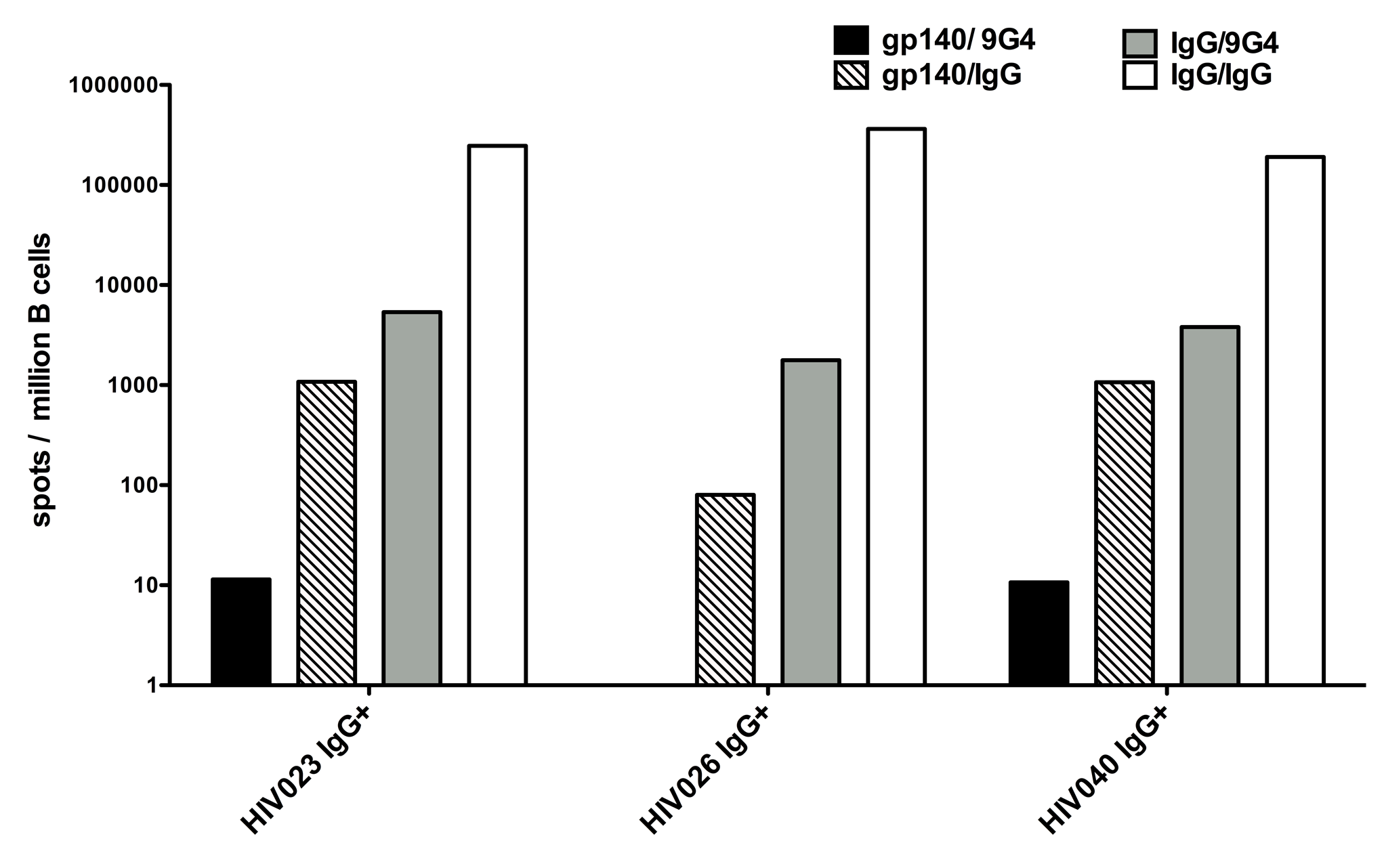

Supplement: Figure S3 — Detection of 9G4+ IgG+ gp140 reactive memory B cells. PBMC were obtained from three HIV patients and IgG+ B cells were isolated and cultured with CpG+IL-2 for 4 days for the generation of antibody-secreting cells. EliSpots were performed to identify total IgG, total 9G4, total IgG gp140, and 9G4+ gp140 specific antibody-secreting cells. EliSpot coating/detection antibody combinations indicated. (TIF) [file pone.0035356.s003.tif]

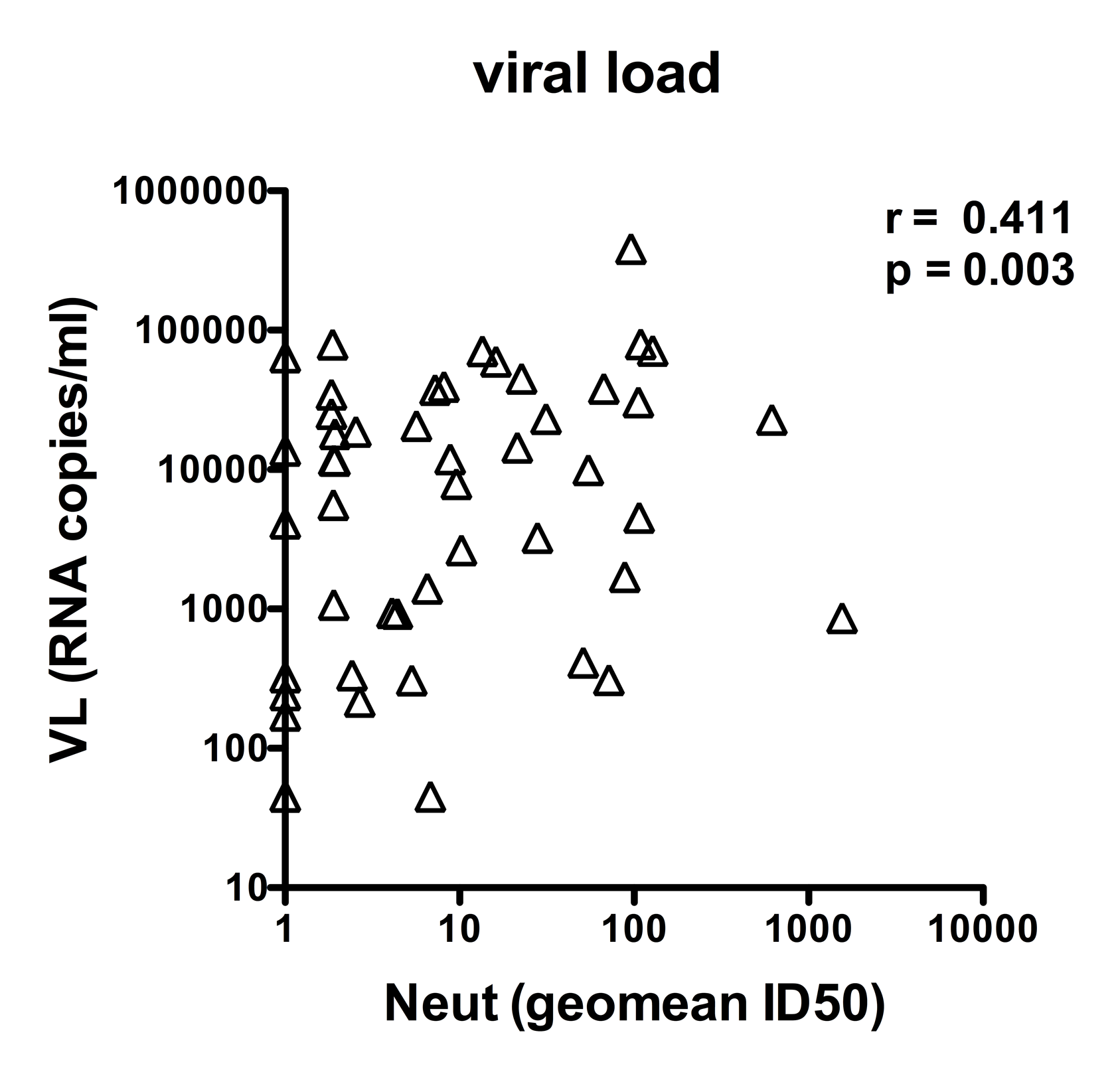

Supplement: Figure S4 — Correlation of HIV serum broadly neutralizing activity and viral load. HIV neutralizing activity of serum against a panel of five Tier II isolates was determined by TZMbl assay and geometric mean ID50 presented with HIV VL. Spearmann correlation indicated, each symbol represents a unique patient. (TIFF) [file pone.0035356.s004.tiff]
