# Supplementary material for: 9G4 Autoreactivity Is Increased in HIV-Infected Patients and Correlates with HIV Broadly Neutralizing Serum Activity
Source: PLoS One. 2012 Apr 18;7(4):e35356. doi: 10.1371/journal.pone.0035356 (PMC3329433; doi:10.1371/journal.pone.0035356)
Supplement: Table S1 — HIV serum neutralizing activity. A. Clade B serum neutralizing activity B. Multi-clade serum neutralizing activity. (PDF) [file pone.0035356.s005.pdf]

	ID50 in TZM-bl cells						
	Tier 1	Tier 2					
Sample	SF162.LS	6535.3	QH0692.42	SC422661.8	PVO.4	AC10.29	RHPA4259.7
HIV073-01	>43740	369	1,087	3,565	1,812	3,506	2,492
HIV026-01	25,459	623	721	554	686	517	1,537
HIV067-01	9,102	104	180	802	919	<20	465
015V08002734	4,391	340	53	205	121	35	551
015V06013685	26,878	116	41	153	183	99	261
015V08000983	964	101	125	188	312	46	53
015V08000147	5,945	95	101	154	128	43	139
015V07000297	>43740	443	55	72	27	29	743
015V05001630	2,473	59	60	96	66	85	296
HIV025-01	12,254	143	151	20	119	104	
HIV028-01	21,180	256	298	126	36	<20	60
015V08001411	19,298	105	78	66	21	43	47
015V08000202	5,764	129	78	46	<20	65	34
HIV027-01	3,239	<20	50	83	44	94	38
015V06004009	1,145	122	33	20	<20	<20	792
HIV056-01	25,750	133	<20	61	24	31	29
015V07003516	5,330	167	42	<20	<20	64	30
015V08000862	2,070	46	65	40	<20	38	<20
HIV041-01	11,627	162	91	<20	<20	<20	<20
015V07005002	>43740	187	31	<20	<20	<20	21
015V06006687	12,479	139	85	<20	<20	<20	<20
015V06006792	4,839	52	77	28	<20	<20	<20
015V07003500	3,197	45	22	50	<20	22	<20
015V08000082	>43740	137	<20	<20	<20	<20	41
HIV066-01	4,154	49	30	<20	<20	37	<20
HIV037-01	8,904	130	33	<20	<20	<20	<20
015V05006388	5,723	38	45	21	<20	<20	<20
015V06006540	309	38	21	<20	25	<20	<20
015V07009464	29,893	36	46	<20	<20	<20	<20
015V07006691	13,099	40	29	<20	<20	<20	<20
015V07010016	1,196	107	<20	<20	<20	<20	<20
HIV068-01	11,906	83	<20	<20	<20	<20	<20
015V07010702	339	<20	27	<20	<20	<20	23
015V06012375	731	<20	23	<20	<20	<20	21
HIV021-01	306	<20	30	<20	<20	<20	
015V07010903	444	27	<20	<20	<20	<20	<20
015V08002003	101	<20	25	<20	<20	<20	<20
015V07010670	333	<20	25	<20	<20	<20	<20
015V07000492	6,138	<20	24	<20	<20	<20	<20
015V05002023	1,422	<20	<20	<20	<20	<20	22
015V08002025	1,292	<20	21	<20	<20	<20	<20
015V09001317	2,686	21	<20	<20	<20	<20	<20
HIV070-01	7,142	<20	<20	<20	<20	<20	<20
HIV057-01	5,404	<20	<20	<20	<20	<20	<20
015V06004249	613	<20	<20	<20	<20	<20	<20
015V08003092	203	<20	<20	<20	<20	<20	<20
HIV016-02	128	<20	<20	<20	<20	<20	<20
HIV064-01	110	<20	<20	<20	<20	<20	<20
HIV011-01	21	<20	<20	<20	<20	<20	
015V07006401	<20	<20	<20	<20	<20	<20	<20
HIV071-01	<20	<20	<20	<20	<20	<20	<20

Supplemental Table 1A. Clade B Serum Neutralizing activity

ID50 ≥500 indicated in red, ID50 100-499 indicated in orange, ID50 20-99 indicated in yellow. Black indicates not tested.

			ID50 in TZM-bl cells	
Virus	Clade	Tier	HIV026-01	HIV028-01
SF162.LS	B	1	25,459	21,180
6535.3	B	2	623	256
QH0692.42	B	2	721	298
SC422661.8	B	2	554	126
PVO.4	B	2	686	36
AC10.29	B	2	517	<20
RHPA4259.7	B	2	1,537	60
WEAU-d15.410.787	B	2	631	149
BB1006-11.C3.1601	B	2	620	296
BB1054-07.TC4.1499	B	2	820	<20
700010040.C9.4520	B	2	333	<20
ZM197M.PB7	C	1	444	68
Du156.12	C	2	2,210	206
Du172.17	C	2	1,034	<20
Du422.1	C	2	413	34
ZM214M.PL15	C	2	728	<20
CAP45.2.00.G3	C	2	571	<20
pCenvFs2_Pt1086_B2	C	2	1,193	<20
pCenvFs4_Pt0393_C3	C	2	1,091	<20
pCenvFs2_Pt1176_A3	C	2	930	41
pCenvFs4_Pt2010_F5	C	2	308	<20
Q23.17	A	1	1,657	178
Q842.d12	A	2	2,019	29
Q168.a2	A	2	1,335	<20
Q259.d2.17	A	2	492	<20
Q461.e2	A	2	249	<20
Q769.d22	A	2	1,355	<20

Supplemental Table 1B. Multi-clade Serum Neutralizing activity

ID50 ≥500 indicated in red, ID50 100-499 indicated in orange, ID50 20-99 indicated in yellow. Black indicates not tested.
